# Chronic Illness Associated with Mold and Mycotoxins: Is Naso-Sinus Fungal Biofilm the Culprit?

**DOI:** 10.3390/toxins6010066

**Published:** 2013-12-24

**Authors:** Joseph H. Brewer, Jack D. Thrasher, Dennis Hooper

**Affiliations:** 1 Plaza Infectious Disease and St. Luke’s Hospital, 4320 Wornall Road, Suite 440, Kansas City, MO 64111, USA; 2 Citrus Heights, CA 95610, USA; E-Mail: toxicologist1@msn.com; 3 RealTime Laboratories, Carrollton, TX 75010, USA; E-Mail: dhooper@realtimelab.com

**Keywords:** mycotoxin, biofilm, rhinosinusitis, chronic fatigue syndrome

## Abstract

It has recently been demonstrated that patients who develop chronic illness after prior exposure to water damaged buildings (WDB) and mold have the presence of mycotoxins, which can be detected in the urine. We hypothesized that the mold may be harbored internally and continue to release and/or produce mycotoxins which contribute to ongoing chronic illness. The sinuses are the most likely candidate as a site for the internal mold and mycotoxin production. In this paper, we review the literature supporting this concept.

## 1. Introduction

Exposure to water damaged buildings (WDB) have been associated with numerous health problems that include fungal sinusitis, abnormalities in T and B cells, central and peripheral neuropathy, asthma, sarcoidosis, respiratory infections and chronic fatigue [1,2,3,4,5,6,7,8,9,10,11,12,13,14]. It has been well established that mold and mycotoxins are important constituents of the milieu in WDB that can lead to illness [15,16,17,18,19,20,21,22]. Using a sensitive and specific assay developed by RealTime Laboratories (RTL), we recently published a study linking the presence of aflatoxins (AT), ochratoxin A (OTA) and/or macrocyclic trichothecenes (MT) to chronic fatigue syndrome (CFS) [14]. The specific methods for these assays have been previously published [14]. A significant number of these chronically ill patients were ill for many years, with an average duration of more than seven years (range 2–36). Furthermore, over 90% of the patients gave a history of exposure to a WDB, mold or both. Exposure histories often indicated the WDB/mold exposure occurred many years prior to the mycotoxin testing. Many of these patients have not had recent or current exposure to a WDB or moldy environment. Despite the remote history of exposure, these patients had chronic symptoms and the presence of significantly elevated concentrations of AT, OTA and MT in their urine specimens. The persistence of mycotoxins suggests that there may be an internal source of mold that represents a reservoir for ongoing mold toxins that are excreted in the urine. Otherwise, one would anticipate that the toxins would have cleared over time. Herein, we discuss the concept that the nose and sinuses may be major internal reservoirs where the mold is harbored in biofilm communities and generates “internal” mycotoxins.

## 2. Example Case Studies

### 2.1. Case One

A 71 year old (y.o.) female was first seen in 1989 with a long standing chronic illness that was subsequently diagnosed as CFS. She had been symptomatic since approximately 1970. She met the Fukuda criteria for CFS as published in 1994 [23]. She has remained chronically ill over the years with minimal variation or improvement in symptoms. The patient had reported long standing sinus problems dating back to childhood. She was diagnosed with chronic sinusitis by the mid-1980s. She underwent two nasal/sinus surgeries, the first in 1988 which entailed nasal reconstruction and the second in 2003 with creation of antral windows. This patient continued with chronic sinus symptoms and required nasal/sinus “clean out” by her Ear, Nose and Throat (ENT) physician about every three months. In 1999, she underwent endoscopy by her ENT physician at which time fungal cultures were obtained. These cultures grew a pure growth of *Aspergillus niger*. The environmental history obtained in 2012, indicated remote exposure to WDB and moldy environments in a home in which she previously lived as well as a work building. These exposures would have occurred in the 1960s. In 2012, a urine mycotoxin assay was sent to RTL which came back positive for OTA at a level of 5.9 parts per billion (ppb). *Aspergillus niger* is one of the fungal species known to produce OTA [15,24].

### 2.2. Cases Two and Three

These cases involve a father (41 y.o.) and daughter (8 y.o.) exposed to mold in a water damaged home as previously reported [25]. They developed numerous health problems following exposure including chronic fungal sinusitis that required surgery [25,26].

Father: The endoscopic sinus surgery performed on the father involved turbinate septoplasty, surgical removal of polyps and debridement of affected sinuses. MRI and CT scans revealed mucosal thickening of all sinuses, particularly the frontal, ethmoid and sphenoid sinuses. The right maxillary sinus had nodular opacities. Surgical specimens were sent to RTL to assay for mycotoxins in the specimens. AT was detected at 1.1 ppb. Culture from the sinus tissue grew *Penicillium* species.

Daughter: The endoscopic exam revealed that maxillary, ethmoid, sphenoid and frontal sinuses were edematous, there were enlarged turbinates (4+) and deviated septum to the left. The endoscopic surgery performed on the daughter involved left sphenoidotomy, ethmoidotomy and maxillary sinusotomy. Surgical specimens sent to RTL demonstrated AT level of 1.2 ppb. A culture obtained from the sinuses was positive for *Aspergillus fumigatus* (*A. fumigatus*).

As previously reported both the father and daughter were positive for mycotoxins in the urine and nasal secretions. The father’s specimens showed the following values: urine OTA 18.2 ppb; nasal secretions AT 11.2 ppb and OTA 13 ppb. The daughter’s results were as follows: urine OTA 28 ppb and MT 0.23 ppb; nasal secretions OTA 3.8 ppb and MT 4.68 ppb. Mycotoxin results for both father and daughter are summarized in Table 1.

**Table 1:** Mycotoxin detection in two cases following exposure in WDB.

Patient: Source	AT ^a^	OTA ^a^	MT ^a^
Father: Sinus Tissue	1.1	NF ^b^	NF
Father: Nasal Secretions	11.2	13	NF
Father: Urine	NF	18.2	NF
Daughter: Sinus Tissue	1.2	NF	NF
Daughter: Nasal Secretions	NF	3.8	4.68
Daughter: Urine	NF	28	0.23

Notes: ^a:^ ppb; ^b:^ Not Found.

## 3. Chronic Rhinosinusitis (CRS)

The nose and paranasal sinuses virtually always harbor numerous fungal species. In a study done at the Mayo Clinic by Ponikau *et al*., numerous types of fungi were recovered from the sinuses of CRS and normal control patients [27]. Amongst the species recovered, many have the potential to produce mycotoxins including *Aspergillus* (*flavus*, *niger*, *fumigatus*, *versicolor*), *Chaetomium*, *Fusarium*, *Penicillium* and *Trichoderma*. This group also found “fungal elements (hyphae, destroyed hyphae, conidiae and spores)” in 82 of 101 (81%) of the surgical specimens from the sinuses. Similarly, Braun *et al*. studied 92 CRS patients and 23 healthy control subjects. Positive cultures for fungi from nasal mucous were found in 91% of CRS patients and 91% of the controls [28]. Fungi and eosinophilic mucin were the markers of sinus involvement in the CRS patients. The species of fungi were very similar to the Mayo study, including potential toxin producing fungi (*Aspergillus*, *Penicillium*, *Chaetomium*, *Trichoderma*). Additionally, of 37 surgical cases, 75% had fungal elements (hyphae and spores) on histological examination. In this paper, the authors state “we conclude that nearly everybody has fungi in his or her nose.” Between the two studies, the total number of different fungal genera identified was 66. Fungal DNA in the sinuses has been identified by quantitative polymerase chain reaction (Q-PCR) of nasal brushings [29]. Similar to the studies noted above, potential mycotoxin producing fungal species were found in the nasal brushings with this method of testing. The species present in the nasal brushings were similar to species found by Q-PCR testing of dust samples in their homes. In another study of CRS, fungal DNA was present in tissue specimens taken from patients with polyploid CRS who underwent surgery [30]. Two PCR primer sets were utilized; one was panfungal and the other specific for *Alternaria*. Fungal DNA was found in all 27 of the CRS patients with both primers. In surgical specimens from healthy controls, the panfungal DNA was positive in 10 of 15 cases but all were negative for the *Alternaria* DNA. Studies have also shown that pre-digestion of tissue slides with trypsin before staining dramatically improves identification of fungi by immunofluorescence as does as PCR-DNA analysis [30,31].

## 4. Detection of Mycotoxins in Invasive Aspergillosis: Humans and Animals

Gliotoxin was detected in the sera of cancer patients with invasive aspergillosis (IA) ranging from 65 to 785 ng/mL. It was also detected in the lungs (3976 ± 1662 µg/g) and in sera (36.5 ± 30.28 ng/mL) of mice with experimentally induced IA [32]. Wild and domestic animals have been reported with IA. Gliotoxin was detected in the lungs of wild birds at 0.1–0.45 mg/kg; an infected bovine udder at 9.2 mg/kg; and turkey poults exceeding 6 ppm in infected tissues [33,34,35]. Moreover, aflatoxin B_1_, B2 and M were detected in the lungs and skin of a patient who died from an invasive infection of *A. flavus* [36]. Aflatoxin B, ranging from 2.0 to 170 µg/g, was recovered in silkworms infected with *A. flavus* [37]. These observations demonstrate that *Aspergillus* species produced mycotoxins in the infectious state in humans and animals. Biofilms may play an important role in that there are up regulated secondary metabolite enzyme pathways in the production of mycotoxins in IA and other mycoses [38,39]. This is discussed further in Section 9. 

## 5. Urine Mycotoxins in CRS Patients

In a study of CRS patients (*n* = 79) by Dennis *et al*., eight patients underwent urine mycotoxin testing for MT that were sent to RTL [2]. Of the eight specimens tested for MT, seven (87%) were positive. Lieberman *et al*. studied 18 patients with CRS. Mycotoxins were detected in urine assays in four of 18 (22%) at 2X the standard deviation above the limit of detection (all were ochratoxin) [40].

## 6. Detection of Mycotoxins from Nasal Washings, Sera and Tissues

Hooper *et al*. found mycotoxins in nasal washings and other tissues of mold exposure cases [41]. The most frequently recovered mycotoxins were MT, found in 44% of the nasal washing specimens, whereas AT were present in 17% of these cases. All nasal washings were negative for mycotoxins in the healthy controls (*n* = 27). In a study of a family exposed to mold in a water damaged home with AT, OTA and MT in environmental samples, nasal washings were positive for mycotoxins (AT, OTA, MT) in three of three family members in which nasal washings were tested [25]. All three cases had positive urine mycotoxins, as well. The specifics of the father and daughter are discussed above in Section 2. Interestingly, in two of the cases, the MT levels recovered from the nasal washings were higher than the urine levels. Between the two studies cited above, AT, OTA and MT have all been demonstrated in nasal washings of patients with clinical illnesses and exposure to a WDB and/or mold. However, mycotoxins were not found in nasal washings of a healthy control population. The results from studies of direct fungal isolation and mycotoxins are summarized in Table 2.

Other positive findings for the presence of mycotoxins in various tissues include the following: MT in sera of individuals exposed in a WDB; breast milk, placenta, umbilical cord and tissues (sinus) from family members exposed to a water damaged home [25,42]. Goats that had *Stachybotrys chartarum* (*S. chartarum*) spores instilled into their trachea were also positive for MT. Although MT cleared from the sera in 24 h, mycotoxins were present at 72 h post installation in the lungs, spleen and lymph nodes [43]. Since *Stachybotrys* is not considered a human pathogen, the uptake of the MT probably occurs from the lysis of spores and/or from other particulate matter. In addition, the detection of MT in lung, spleen and lymph nodes indicates peripheral organ storage has occurred.

**Table 2:** Presence of fungi and mycotoxins in healthy individuals, Chronic Rhinosinusitis (CRS) patients and mold exposure cases.

Study	Type of patients	Fungi present sinuses	Potential mycotoxin producing fungi in sinuses	Urine mycotoxins present	Nasal washing mycotoxins present
Ponikau [27]	Normal	Yes	Yes	ND ^b^	ND
Ponikau	CRS ^a^	Yes	Yes	ND	ND
Braun [28]	Normal	Yes	Yes	ND	ND
Braun	CRS	Yes	Yes	ND	ND
Murr [29]	CRS	Yes	Yes	ND	ND
Dennis [2]	CRS	ND	ND	Yes	ND
Lieberman [40]	CRS	ND	ND	Yes	ND
Hooper [41]	Normal	ND	ND	No	No
Hooper	Mold exposure	ND	ND	Yes	Yes
Thrasher [25]	Mold exposure	Yes	Yes	Yes	Yes

Notes: a: Chronic rhinosinusitis; b: Not done.

## 7. Indoor Microbes and Their Fragments

Mycotoxins (AT, OTA, MT) produced by several species of mold have been identified in water-damaged indoor environments [15,16,17,18,19,21,25]. They have been detected in the sera, urine and tissues of individuals with illness associated with exposure to microbes in these contaminated environments [2,3,14,25,40,41,42]. Whereas species of *Aspergillus* and *Penicillium* have been demonstrated in the nasal cavity and sinuses of individuals with CRS, accounting for the probable source of AT and OTA, the detection of MT appears to be somewhat of an enigma. *Trichoderma* has been found in the sinuses and does produce MT [27,28]. However, *S. chartarum* does not germinate and grow in animal tissues [22]. Furthermore, *S. chartarum* has not been recovered from patients with CRS either by culture or Q-PCR, although it is present in the dust of homes with affected occupants [15,16,17,18,19,20,21,22]. Since, *S. chartarum* does not readily shed its spores, what are the possible explanations for the detection of its mycotoxins in humans exposed to damp-indoor environments? We will briefly review the literature regarding the release of ultrafine particles (nanoparticles) by colonies of mold commonly present in damp-indoor spaces.

*S. chartarum*, other molds and bacteria produce large quantities of fine (nano range) fragments (0.03 to 0.3 microns) when compared to airborne spore counts [44,45,46,47,48,49]. The number of fine fragments is at least 500 times greater than the spore counts [46,47,48]. The respiratory deposition of these fine fungal fragments is 230 times that of spores including the anterior nasal region [46]. Furthermore, the fragments (small particulates) produced by *Stachybotrys* contain MT while other mold fragments (e.g., *Aspergillus* and *Penicillium*) contain antigens and toxins as determined by ELISA testing [19,42,44,45]. Thus, fungal fragments, which contain MT, as well as other mycotoxins and antigens, are inhaled and most likely deposited in the nasal cavity and sinuses. The fungal fragments are not detected by spore counts, in culture or even Q-PCR [44,45,46,47,48,49]. It has been recommended that the role of the fine particulates shed by mold and bacteria needs to be evaluated for contribution to the health problems of the exposed, rather than relying upon airborne mold spore counts [44,45,46,47,48,49]. These fine particulates may contribute to the colonization of the nose and sinuses which may be a particularly significant issue with *S. chartarum*. 

## 8. Antifungal Therapy Directed at the Sinuses

In a study of treatment of patients with an intranasal antifungal agent (amphotericin B solution), Ponikau *et al*. showed significant improvements in several clinical parameters (symptoms, endoscopic findings and CT scanning of the sinuses) in CRS patients [50,51,52,53]. The authors concluded that reducing the amount of fungal antigen with the antifungal therapy led to clinical improvements. Varied results from studies of CRS treatments may be due to the fact that CRS can result from infection by bacteria, invasive mold, mold colonization in the presence of biofilms, the extent of sinus involvement (e.g., sphenoid sinuses) or a combination of factors [38,39,52,53]. Surgical debridement is also a common treatment in CRS [1,2,53]. Identifying specific fungal organisms in CRS caused by mold requires specific fungal staining methods to identify hyphae in sinus specimens or identification of mold by Q-PCR [30,31,54,55]. Treatment of fungal CRS may require the use of oral antifungals, as well as intranasal sprays with antifungal activity, depending upon the improvement of individual patient condition [56]. In addition, biofilms, antifungal shelf-life and antifungal resistance must be considered as other variables in effectiveness of treatment [57,58,59,60,61,62,63,64].

A recent study of mold exposed patients (*n* = 25) with a variety of systemic symptoms was presented [63]. The vast majority of the patients were positive for mycotoxins in the urine. The patients were treated with intranasal amphotericin B with or without systemic antifungals which represented biofilm focused therapy. The patients were monitored before and after treatment. Ninety per cent of the patients had a dramatic decrease in their systemic symptoms, including neurological conditions of tremor, ataxia and vertigo, among others [63].

## 9. Role of Biofilm

Biofilms are produced by bacteria and molds and are present in CRS. We will briefly review the key aspects of biofilms and their role in resistance of the microorganisms to antifungal treatment. Often the failure of such treatments lead to surgical intervention [1,2,53,61,64].

**Figure 1:**
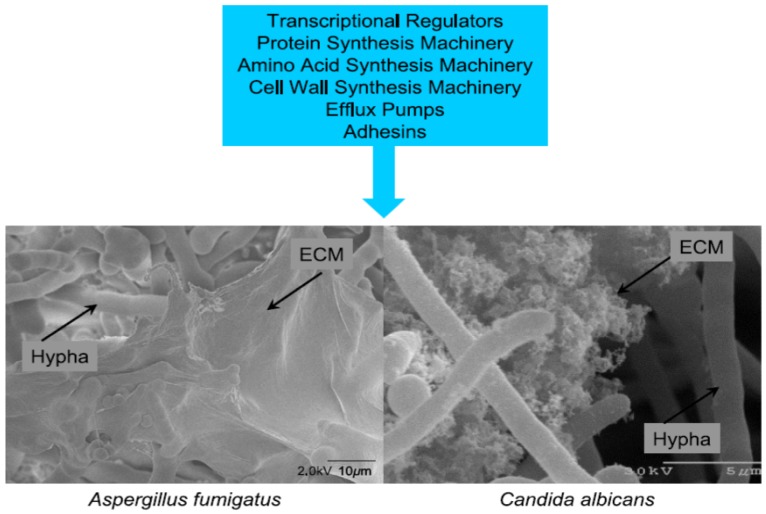
Common features of fungal biofilms. Gene expression has been compared between planktonic and biofilm cells of both *A. fumigatus* and *Candida albicans.* The major categories of genes up regulated in biofilms are summarize in the blue box. The photos depict the biofilm of *A. fumigatus* and *C. albicans*. The missing ingredient of the blue box is the up regulation of secondary metabolite pathways as demonstrated *in vitro* by Bruns *et al*. [38]. Permission to publish this figure was given by Dr. Fanning and Mitchell [39].

Briefly, biofilms are complex surface-associated populations of microorganisms embedded in an extracellular matrix (ECM) that possess distinct phenotypes compared to planktonic (free living) organisms. *In vitro* and *in vivo* observations have revealed the morphology and matrix of fungal biofilms [60,61,62,64]. Epithelial cells isolated from sinuses of CRS patients and controls were grown to a confluent monolayer *in vitro* and then infected with *A. fumigatus* under static and flow conditions [62]. The formation of the biofilm occurred in five stages: (1) conidial attachment to epithelial cells; (2) hyphal proliferation; (3) extracellular matrix (ECM); (4) hyphal parallel packing and cross linking; and (5) channel/pore formation. Biomass of the film was greater in flow *versus* static conditions [62]. The architecture of the biofilm was similar to that reported from *in vivo* CRS conditions as shown in Figure 1 [39]. The fungal ECM consists of polysaccharides (galactomannan, β-d-glucans, lipopolysaccharides), among other extracellular proteins, exotoxins, melanin, hydrophobins, exotoxins, monosaccharides and probably mycotoxins [39,59,64,65,66,67,68]. In this regard, biofilm cells have phenotypes and gene expressions distinct from the planktonic cells. Gene expression of a variety of pathways can be up or down regulated in the biofilm cells when compared to the planktonic phenotypes [38,39,69]. For example, over 3,000 differentially regulated genes have been identified under the two conditions [70]. Some of the genes impart antifungal resistance or up regulation of secondary metabolite pathways [38,39].

Gliotoxin produced by *A. fumigatus* was detected in an *in vitro* biofilm model. The proteins of the gliotoxin secondary metabolite pathway were up regulated in the biofilm cultures [38]. The ability of *A. fumigatus* to form biofilms is considered an important factor in invasive disease [67,68,70,71,72,73]. Thus, the presence of mycotoxins in human tissues and body fluids with invasive mycoses probably occurs. The gliotoxin detected in the sera of cancer patients and in various animals with invasive IA was reviewed in Section 4. Moreover, the detection of aflatoxin B_1_, B_2_ and M were detected in the lungs and skin of a patient who died from an IA was also reviewed in Section 4. These observations demonstrate that *Aspergillus* species produced mycotoxins in the infectious state in humans and animals. Biofilm may be a factor in up regulated secondary metabolite enzyme pathways in the production of mycotoxins in IA and other mycoses.

There is an apparent interaction and possible synergy between bacteria and fungi in biofilm development and survival. In a sheep model, bacteria appear to induce epithelial damage that promotes fungal biofilm formation by *A. fumigatus.* Co-inoculation of *Staphylococcus aureus* (*S. aureus*) and *A. fumigatus* into sheep sinuses resulted in an 80% formation of biofilms *versus* 10% with *A. fumigatus* inoculation alone [74,75]. Such interaction may provide better surface adherence and ECM formation. In a study by Foreman *et al*., the microbiology of biofilms was studied in CRS patients using a sensitive fluorescent *in situ* hybridization (FISH) assay [60]. 36 of 50 CRS patients had biofilms compared to 0 of 10 controls. *S. aureus* was the most common bacterial isolate found. Fungi (using a panfungal probe) were found in 11 of 50 cases. Of these 11 fungal biofilms, seven also demonstrated *S. aureus* biofilms. In another publication, *Haemophilus influenzae* produced less severe disease than *S. aureus* [65]. *S. aureus*, coagulase negative staphylococci (CNS) and other bacteria are frequently found in the sinuses, both in controls and CRS patients [76,77,78,79,80,81,82]. CNS has clearly been demonstrated to produce biofilm which represents a major pathogenic mechanism for these bacteria in certain clinical settings [80,81]. Since *S. aureus*, CNS and other bacteria frequently occur in the sinuses and commonly form biofilm, this may potentially represent another significant co-pathogen for fungal biofilm formation.

The biofilm confers considerable protection for the organisms including resistance to host defenses and antifungal treatments [38,39,64,83,84]. ECM acts as a physical barrier between the embedded fungal cells and clinically useful antifungal agents, thus leading to ongoing colonization of fungi in the sinuses despite maximal treatment [39,64,83]. Biofilm may allow for chronic persistence of fungi in the nose and sinuses and make treatments more difficult. Although the efficacy of antifungal treatments has been questioned in biofilm, amphotericin B has worked reasonably well in clinical settings and in biofilm models [50,63,84,85]. This may be especially the case for higher concentrations of amphotericin B, which can be used in sinus irrigation since there is no systemic absorption [50,51,52,84]. A combination of amphotericin B with voriconazole and caspofungin was tested on *A. fumigatus* from early to late stages of colony growth. The combination was effective during early growth, while amphotericin B alone was most effective in the later stages of mycelial growth [84,85].

Given the role of bacterial pathogens in fungal sinus biofilm (e.g., *S. aureus*), antibacterial therapy may be a helpful adjunct. For example, mupirocin was shown to be effective in the post surgical treatment of recalcitrant CRS [82].

Other agents such as N-acetyl cysteine (NAC) and EDTA may assist with disruption of biofilm and enhance the activity of antifungal and antibacterial drugs [86].

Therapies directed at the fungal biofilm may be promising potential interventions for patients with chronic illness secondary to mycotoxins. Examples of such therapies could include agents to disrupt biofilm (e.g., intranasal EDTA) and intranasal antifungal administration (e.g., amphotericin B).

## 10. Conclusions

(1) Indoor water-damaged environments contain a variety of mold and bacterial species that produce mycotoxins, volatile organic compounds, exotoxins and other metabolites that are present in the dust, furnishings and air [15,16,17,18,19,20,21,22];
(2) The occupants of these environments experience chronic adverse health effects that range from upper and lower respiratory disease, central and peripheral neurological deficits, chronic fatigue type illness, among others [1,2,3,4,5,6,7,8,9,10,11,12,13,14];
(3) Patients that remain chronically ill (e.g., CFS) after exposure to WDB and/or mold, very commonly demonstrate mycotoxins in the urine [14,25,40,41]. Many of these patients have remained chronically ill despite leaving the moldy environment several years previous to the urine testing [14]. This suggested to us that there may well be an internal presence of toxin producing mold. We raised the question, where was the mold located in the body? Herein, we have reviewed the medical literature as it relates to the presence of fungi/mold in the nose and sinuses;
(4) We reviewed data for three patients with chronic illness who required surgery for chronic fungal rhinosinusitis. Mycotoxin testing revealed the presence of AT, OTA, and MT in nasal secretions, urine and tissues samples (Table 1 and Table 2) as reported herein and by others [2,3,14,25,40,41,42]. Additionally, fungal organisms were recovered in cultures from the sinuses in these three cases including *Aspergillus niger*, *Aspergillus fumigatus* and *Penicillium*;
(5) Humans and animals with IA have gliotoxin and aflatoxins in their sera and tissues [32,33,34,35,36,37]. These observations suggest that *Aspergillus* species produce mycotoxins during IA. In addition, after intratracheal administration of *Stachybotrys* spores, animals were found to have MT in their lungs, spleen and lymph nodes at 72 h after treatment [42]. Also, storage of mycotoxins occurs in variety of tissues [36,41,42];
(6) Fungal species can be found in the sinuses of normal, healthy individuals, as well as CRS patients [27,28,55]. Species that have been recovered include those that have the capacity to produce mycotoxins. Additionally, mycotoxins (AT, OTA and MT) have been recovered from nasal washings in patients exposed to a moldy environment, however they were not found in nasal washings of healthy individuals [41];
(7) The fungi that are present in the sinuses are in biofilm communities which allows for chronic persistence [39,60,61,65,66,67,68]. This would explain the chronic nature of the fungi/mold in the sinuses and explain the difficulty in treatment [39,64,83]. However, despite that, studies have demonstrated success with treating patients with intranasal amphotericin B. This was shown in both CRS patients and those with chronic illness following mold exposure [50,51,63]. Amphotericin B has been shown to have superior activity in biofilm models as opposed to other antifungal agents [50,51,84];
(8) Fungal fragments from 0.03 to 0.3 microns are shed from fungal colonies known to contain antigens and toxins [44,45,46,47,48]. Fine particulates shed by *Stachybotrys* contain MT [18,19]. The fragments are readily deposited in the nasal cavity [46]. MT have been detected in the sera of occupants exposed to *Stachybotrys* [42];
(9) Prior exposure to toxic mold and mycotoxins may represent an important feature of chronically ill patients such as CFS as well as those with CRS. An internal reservoir of toxin producing mold (e.g., sinuses) that persists in biofilms could produce and release mycotoxins. This model of fungal persistence may help explain these chronic illnesses and represent a potential new understanding of mechanisms of disease that can be treated and/or lessened.

## References

[1] Dennis D.P. (2003). Chronic defective T-cells responding to superantigens, treated by reduction of fungi in the nose and air. Arch. Environ. Health.

[2] Dennis D.P., Roberson D., Curtis L., Black J. (2009). Fungal exposure endocrinopathy with growth hormone deficiency; Dennis-Robertson syndrome. Toxicol. Ind. Health.

[3] Rea W.J., Didriksen N., Simon T.R., Pan Y., Fenyves E.J., Griffiths G. (2003). Effects of toxic exposure to mold and mycotoxins in building-related illnesses. Arch. Environ. Health.

[4] Campbell A., Thrasher J.D., Gray M.R., Vojdani A. (2004). Mold and mycotoxins: Effects on the neurological and immune systems in humans. Adv. Appl. Microbiol..

[5] Gray M.R., Thrasher J.D., Crago R., Madison R.A., Arnold L., Campbell A.W., Vojdani A. (2003). Mixed mold mycotoxicosis: Immunological changes in humans following exposure to water damaged buildings. Arch. Environ. Health.

[6] Kilburn K.H. (2009). Neurobehavioral and pulmonary impairment in 105 adults with indoor exposure to molds compared to 100 exposed to chemicals. Toxicol. Ind. Health.

[7] Empting L.D. (2009). Neurologic and neuropsychiatric syndrome features of mold and mycotoxin exposure. Toxicol. Ind. Health.

[8] Park J.H., Cox-Ganser J.M. (2011). Mold exposure and respiratory health in damp indoor environments. Front. Biosci..

[9] Fisk W.J., Eliseeva E.A., Mendell M.J. (2010). Association of residential dampness and mold with respiratory tract infections and bronchitis: A meta-analysis. Environ. Health.

[10] Park J.H., Kreiss K., Cox-Ganser J.M. (2012). Rhinosinusitis and mold as risk factors for asthma symptoms in occupants of a water-damaged building. Indoor Air.

[11] Tercelj M., Salobir B., Harlander M., Rylander R. (2011). Fungal exposure in homes of patients with sarcoidosis—An environmental exposure study. Environ. Health.

[12] Laney A.S., Cragin L.A., Blevins L.Z., Sumner A.D., Cox-Ganser J.M., Kreiss K., Moffatt S.G., Lohff C.J. (2009). Sarcoidosis, asthma and asthma-like symptoms among occupants of a historically water-damaged office building. Indoor Air.

[13] Chester A.C., Levine P. (1994). Concurrent sick building syndrome and chronic fatigue syndrome: Epidemic neuromyasthenia revisited. Clin. Infect. Dis..

[14] Brewer J.H., Thrasher J.D., Straus D.C., Madison R.A., Hooper D. (2013). Detection of mycotoxins in patients with chronic fatigue syndrome. Toxins.

[15] Polizzi V., Delmulle B., Adams A., Moretti A., Susca A., Picco A.M., Rosseel Y., Kindt R., van Bocxlaer J., de Kimpe N. (2009). JEM Spotlight: Fungi, mycotoxins and microbial volatile organic compounds in mouldy interiors from water-damaged buildings. J. Environ. Monit..

[16] Smoragiewicz W., Cossette B., Boutard A., Kryzvstyniak K. (1993). Trichothecene mycotoxins in the dust of ventilation systems in office buildings. Int. Arch. Occup. Environ. Health.

[17] Täubel M., Sulyok M., Vishwanath V., Bloom E., Turunen M., Järvi K., Kauhanen E., Krska R., Hyvärinen A., Larsson L. (2011). Co-occurrence of toxic bacterial and fungal secondary metabolites in moisture-damaged indoor environments. Indoor Air.

[18] Gottschalk C., Bauer J., Meyer K. (2008). Detection of Satratoxin G and H in indoor air from a water-damaged building. Mycopatholgia.

[19] Brasel T.L., Martin J.M., Carriker C.G., Wilson S.C., Straus D.C. (2005). Detection of airborne *Stachybotrys chartarum* macrocyclic trichothecenes in the indoor environment. Appl. Environ. Microbiol..

[20] Thrasher J.D., Crawley S. (2009). The biocontaminants and complexity of damp indoor spaces: More than meets the eyes. Toxicol. Ind. Health.

[21] Straus D.C. (2009). Molds, mycotoxins, and sick building syndrome. Toxicol. Ind. Health.

[22] Pestka J.J., Yike I., Dearborn D.G., Ward M.D., Harkema J.R. (2008). *Stachybotrys chartarum*, trichothecenes mycotoxins, and damp building-related illness: New insights into a public health enigma. Toxicol. Sci..

[23] Fukuda K., Strauss S.E., Hickie I., Sharpe M.C., Dobbins J.G., Komaroff A. (1994). The chronic fatigue syndrome: A comprehensive approach to its definition and study. Ann. Intern. Med..

[24] Ostry V., Malir F., Ruprich J. (2013). Producers and important dietary sources of ochratoxin A and citrinin. Toxins.

[25] Thrasher J.D., Gray M.R., Kilburn K.H., Dennis D., Yu A. (2012). A water-damaged home and health of occupants: A case study. J. Environ. Public Health.

[26] Dennis D.P. (2010). Personal Communication.

[27] Ponikau J.U., Sherris D.A., Kern E.B., Homeburger H.A., Frigas E., Gaffey T.A., Roberts G.D. (1999). The diagnosis and incidence of allergic fungal sinusitis. Mayo Clin. Proc..

[28] Braun H., Buzina W., Freudenschuss F., Beham A., Stammberger H. (2003). “Eosinophilic fungal rhinosinusitis”: A common disorder in Europe?. Laryngoscope.

[29] Murr A.H., Goldberg A.N., Pletcher S.D., Dillehay K., Wymer L.J., Vesper S.J. (2012). Some chronic rhinosinusitis patients have elevated populations of fungi in their sinuses. Laryngoscope.

[30] El-Morsy S.M., Khafagy Y.W., El-Naggar M.M., Beih A.A. (2010). Allergic fungal rhinosinusitis: Detection of fungal DNA in sinus aspirate using polymerase chain reaction. J. Layrngol. Otol..

[31] Guo C., Ghadersohi S., Kephart G.M., Laine R.A., Sherris D.A., Kita H., Ponikau J.U. (2012). Improving detection of fungi in eosinophilic mucin: Seeing what we could not see before. Otolaryngol. Head Neck Surg..

[32] Lewis R.E., Wiederhold N.P., Chi J., Han X.Y., Komanduri K.V., Kontoyiannis D.P., Prince R.A. (2005). Detection of gliotoxin in experimental and human aspergillosis. Infect. Immun..

[33] Korbel R., Bauer J., Gedek B. (1998). Pathologico-anatomic and mycotoxicologic studies of aspergillosis in birds. Tierarit Prax.

[34] Bauer J., Gaareis M., Bott A., Gedek B. (1989). Isolation of a mycotoxin (gliotoxin) from a bovine udder infected with *Aspergillus fumigatus*. J. Med. Vet. Mycol..

[35] Richard J.L., Debey M.C. (1995). Production of gliotoxin during pathogenic state in turkey poults by *Aspergillus fumigatus*. Fresneius Mycopathol..

[36] Matsumara M., Mori T. (1998). Detection of aflatoxins in autopsied materials from a patient infected with *Aspergillus flavus*. Jpn. J. Med. Mycol..

[37] Ohtomo T., Murkakoshi S., Sugiyama S., Kurata H. (1975). Detection of aflatoxin B1 in silkworm larvae attached by an *Aspergillus flavus* isolate from a sericultural farm. Appl. Microbiol..

[38] Bruns S., Seidler M., Albrecht D., Salvenmoser S., Remme N., Hertweck C., Brakhage A.A., Kniemeyer O., Müller F.M. (2010). Functional genome profiling of *Aspergillus fumigatus* biofilm reveals enhanced production of the mycotoxin gliotoxin. Proteomics.

[39] Fanning S., Mitchell A.P. (2012). Fungal biofilms. PLoS Pathog..

[40] Lieberman S.M., Jacobs J.B., Lebowitz R.A., Fitzgerald M.B., Crawford J., Feigenbaum B.A. (2011). Measurement of mycotoxins in patients with chronic rhinosinusitis. Otolaryngol. Head Neck Surg..

[41] Hooper D.G., Bolton V.E., Guilford F.T., Straus D.C. (2009). Mycotoxin detection in human samples from patients exposed to environmental molds. Int. J. Mol. Sci..

[42] Brasel T.L., Campbell A.W., Demers R.E., Ferguson B.S., Fink J., Vojdani A., Wilson S.C., Straus D.C. (2004). Detection of trichothecene mycotoxins in sera from individuals exposed to *Stachybotrys chartarum* in indoor environments. Arch. Environ. Health.

[43] Layton R.C., Purdy C.W., Jumper C.A., Straus D.C. (2009). Detection of macrocyclic trichothecene mycotoxins in a *caprine* (goat) tracheal instillation model. Toxicol. Ind. Health.

[44] Gorny R.L., Reponen T.L., Willeke K., Schmechel D., Robine E., Boissier M., Grinshpun S.A. (2002). Fungal fragments as indoor air biocontaminants. Appl. Environ. Microbiol..

[45] Gorny R.L. (2004). Filamentous microorganisms and their fragments in indoor air—A review. Ann. Agric. Environ. Med..

[46] Cho S.-H., Seo S.-C., Schmechel D., Grinshpun A.G., Reponen T. (2005). Aerodynamic characteristics and respiratory deposition of fungal fragments. Atmos. Environ..

[47] Reponen T., Seo S.-C., Grimsley F., Lee T., Crawford C., Grinshpun S.A. (2007). Fungal fragments in moldy houses: A field study in homes in New Orleans and Southern Ohio. Atmos. Environ..

[48] Gorny R.L., Lawniczek-Walczyk A. (2012). Effect of two aerosololization methods on the release of fungal propagules from a contaminated agar surface. Ann. Agric. Environ. Med..

[49] Scott J. An Evolving Architecture: Past, Present & Future and Indoor Microbiology. Proceedings of the Indoor Air Quality Association 15th Annual Meeting and Indoor Air Expos.

[50] Ponikau J.U., Sherris D.A., Hirohito K., Kern E.B. (2002). Intranasal antifungal treatment in 51 patients with chronic rhinosinusitis. J. Allergy Clin. Immunol..

[51] Ponikau J.U., Sherris D.A., Weaver A., Kita H. (2005). Treatment of chronic rhinosinusitis with intranasal amphotericin B: A randomized, placebo-controlled, double-blind pilot trial. J. Allergy Clin. Immunol..

[52] Kern E.B., Sherris D., Stergiou A.M., Katz L., Rosenblatt L.C., Ponikau J. (2007). Diagnosis and treatment of chronic rhinosinusitis: A focus on intranasal Amphotericin B. Ther. Clin. Risk Manag..

[53] Chakrabarti A., Denning D.W., Ferguson B.J., Ponikau J., Buzina W., Kita H., Marple B., Panda N., Vlaminck S., Kauffmann-Lacroix C. (2009). Fungal rhinosinusitis: A categorization and definitional schema addressing current controversies. Laryngoscope.

[54] Siddiqui A., Shah A.A., Bashir S.H. (2004). Craniocerebral aspergillosis of sinonasal origin in immunocompetent patients: Clinical spectrum and outcome of 25 cases. Neurosurgery.

[55] Gosepath J., Brieger J., Vlachtsis K., Mann W.J. (2004). Fungal DNA is present in tissue specimens of patients with chronic rhinosinusitis. Am. J. Pathol..

[56] Gray M.R. (2012). Personal communication.

[57] Bristol-Myers Squibb (2009). Fungizone Product Monograph.

[58] Geronikaki A., Fesatidou M., Kartsey V., Macaey F. (2013). Synthesis and biological evaluation of potent antifungal agents. Curr. Top. Med. Chem..

[59] Kidane Y.H., Lawrence C., Murali T.M. (2013). Computational approaches for discovery of common immunomodulators in fungal infections: Towards broad-spectrum immunotherapeutic interventions. BMC Microbiol..

[60] Foreman A., Psaltis A.J., Tan L.W., Wormald P.J. (2009). Characterization of bacterial and fungal biofilms in chronic rhinosinusitis. Am. J. Rhinol. Allergy.

[61] Pintucci J.P., Corno S., Garotta M. (2010). Biofilms and infections of the upper respiratory tract. Eur. Rev. Med. Pharmacol. Sci..

[62] Singhal D., Baker L., Wormold P.J., Tan L.W. (2011). *Aspergillus fumigatus* biofilm on primary human sinonasal epithelial culture. Am. J. Rhinol. Allergy.

[63] Rini J.F., Grant I.H. Neurological Disease after Mold Exposure, Immune Risks & Response to Biofilm-Focused Antifungal Therapy. Proceedings of the 52nd Annual Interscience Conference on Antimicrobial Agents and Chemotherapy Conference.

[64] Ramage G., Rajendran R., Sherry L., Williams C. (2012). Fungal biofilm resistance. Int. J. Microbiol..

[65] Fey P.D. (2010). Modality of bacterial growth presents unique targets: How do we treat biofilm-mediated infections. Curr. Opin. Microbiol..

[66] Foreman A., Wormald P.J. (2010). Different biofilms, different disease? A clinical outcomes study. Laryngoscope.

[67] Loussert C., Schmitt C., Prévost M.C., Balloy V., Fadel E., Philippe B., Kauffmann-Lacroix C., Latjé J.P., Beauvais A. (2010). The *in vivo* biofilm composition of *Aspergillus fumigatus*. Cell. Microbiol..

[68] Beauvais A., Schmidt C., Guadagnini S., Roux P., Perret E., Henry C., Paris S., Mallet A., Prévost M.C., Latejé J.C. (2007). An extracellular matrix glues together the aerial-grown hyphae of *Aspergillus fumigatus*. Cell. Microbiol..

[69] Hung C., Zhou Y., Pinkner J.S., Dodson K.W., Crowley J.R., Heuser J., Chapman M.R., Hadjifrangiskou M., Henderson J.P., Hultgren S.J. (2013). *Escherichia coli* biofilms have organized complex extracellular matrix structure. MBio.

[70] Gibbons J.G., Beauvais A., Beau R., McGary L., Latgé J.P., Rokas A. (2012). Global transcriptome changes underlying colony growth in the opportunistic human pathogen *Aspergillus fumigatus*. Eukaryot. Cell.

[71] Shopova I., Bruns S., Thywissen A., Kniemeyer O., Brakhage A.A., Hillmann F. (2013). Extrinsic extracellular DNA leads to biofilm formation and colocalizes with matrix polysaccharides in the human pathogenic fungus *Aspergillus fumigatus*. Front. Microbiol..

[72] Muller F.M., Seider M., Beauvais A. (2011). *Aspergillus fumigatus* in the clinical setting. Med. Mycol..

[73] Kaur S., Singh S. (2013). Biofilm formation by *Aspergillus fumigatus*. Med. Mycol..

[74] Boase D., Jervis-Bardy J., Cieland E., Pant H., Tan L., Wormald P.J. (2013). Bacterial-induced epithelial damage promotes fungal biofilm formation in a sheep model of sinusitis. Int. Forum Allergy Rhinol..

[75] Boase S., Valentine R., Singhal D., Tan L.W., Wormald P.J. (2011). A sheep model to investigate the role of fungal biofilms in sinusitis: Fungal and bacterial synergy. Int. Forum Allergy Rhinol..

[76] Tan N.C., Tran H.B., Foreman A., Jardeleza C., Vreudge S., Wormold P.J. (2012). Identifying intracellular *Staphylococcus aureus* in chronic rhinosinusitis: A direct comparison of techniques. Am. J. Rhinol. Allergy.

[77] Biel M.A., Brown C.A., Levinson R.M., Garvis G.E., Paisner H.M., Sigel M.E., Tedford T.M. (1998). Evaluation of the microbiology of chronic maxillary sinusitis. Ann. Otol. Rhinol. Laryngol..

[78] Aral M., Keleş E., Okur E., Alpay H.C., Yilmaz M. (2004). The pathogenicity and antibiotic resistance of coagulase-negative Staphylococci isolated from the maxillary and ethmoid sinuses. Rhinology.

[79] Aral M., Keles E., Kaygusuz I. (2003). The microbiology of ethmoid and maxillary sinuses in patients with chronic rhinosinusitis. Am. J. Otolaryngol..

[80] O’Gara J.P., Humphreys H. (2001). *Staphylococcus epidermidis* biofilms: Importance and implications. J. Med. Microbiol..

[81] Mack D., Haeder M., Siemssen N., Laufs R. (1996). Association of biofilm production of coagulase-negative Staphylococci with expression of a specific polysaccharide intracellular adhesion. J. Infect. Dis..

[82] Uren B., Psaltis A., Wormold P.J. (2008). Nasal lavage with mupirocin for the treatment of surgically recalcitrant chronic rhinosinusitis. Laryngoscope.

[83] Ebbens F.A., Scadding G.K., Badia L., Hellings P.W., Jorissen M., Mullol J., Cardesin A., Bachert C., van Zele T.P., Dijkgraaf M.G. (2006). Amphotericin B nasal lavages: Not a solution for patients with chronic rhinosinusitis. J. Allergy Clin. Immunol..

[84] Mowat E., Butcher J., Lang S., Williams C., Ramage G. (2007). Development of a simple model for studying the effects of antifungal agents on multicellular communities of *Aspergillus fumigatus*. J. Med. Microbiol..

[85] Mowat E., Lang S., Williams C., McCulloch E., Jones B., Ramage G. (2008). Phase-dependent antifungal activity against *Aspergillus fumigatus* developing multicellular filamentous biofilms. J. Antimicrob. Chemother..

[86] Venkatesh M., Rong L., Raad I., Versalovic J. (2009). Novel synergisitic antibiofilm combinations for salvage of infected catheters. J. Med. Microbiol..

